# Selective bacterial colonization processes on polyethylene waste samples in an abandoned landfill site

**DOI:** 10.1038/s41598-019-50740-w

**Published:** 2019-10-02

**Authors:** Edoardo Puglisi, Francesco Romaniello, Serena Galletti, Enrico Boccaleri, Alberto Frache, Pier Sandro Cocconcelli

**Affiliations:** 1 Dipartimento di Scienze e Tecnologie Alimentari per la sostenibilità della filiera agro-alimentare, Facoltà di Scienze Agrarie Alimentari ed Ambientali, Università Cattolica del Sacro Cuore, Via Emilia Parmense 84, 29122 Piacenza, Italy; 2 AAT- Advanced Analytical Technologies, Via Majavacca 12, 29017, Fiorenzuola d’Arda, Piacenza, Italy; 3 Dipartimento di Scienze e Innovazione Tecnologica, Università del Piemonte Orientale “A. Avogadro”, Via Michel 11, I-15121 Alessandria, Italy; 4 Dipartimento Scienza Applicata e Tecnologia, Politecnico di Torino, Viale Teresa Michel 5, Alessandria, Italy

**Keywords:** Soil microbiology, Pollution remediation

## Abstract

The microbial colonization of plastic wastes has been extensively studied in marine environments, while studies on aged terrestrial wastes are scarce, and mostly limited to the isolation of plastic-degrading microorganisms. Here we have applied a multidisciplinary approach involving culturomics, next-generation sequencing analyses and fine-scale physico-chemical measurements to characterize plastic wastes retrieved in landfill abandoned for more than 35 years, and to assess the composition of bacterial communities thriving as biofilms on the films’ surfaces. All samples were characterized by different colors but were all of polyethylene; IR and DSC analyses identified different level of degradation, while FT-Raman spectroscopy and X-ray fluorescence further assessed the degradation level and the presence of pigments. Each plastic type harbored distinct bacterial communities from the others, in agreement with the differences highlighted by the physico-chemical analyses. Furthermore, the most degraded polyethylene films were found to host a bacterial community more similar to the surrounding soil as revealed by both α- and β-diversity NGS analyses. This work confirms the novel hypothesis that different polyethylene terrestrial waste samples select for different bacterial communities, and that structure of these communities can be correlated with physico-chemical properties of the plastics, including the degradation degree.

## Introduction

Environmental pollution by plastic materials is a global environmental issue. Recent data show continuous increase in the global production of plastic materials, passing from 322 tons in 2015 to 335 million in 2016^1^. Albeit the last years have shown an increase in the percentage of plastics being recycled (40.9% in 2016), the proportion of plastics being discharged in landfills or not properly disposed is still more than 1/5 of the global annual production^1^. The disposal to landfill and in general the release in the environment is indeed the biggest issue related to plastics, especially because most of the plastics produced belongs to chemical classes that are strongly resistant to degradation processes: polyethylene (PE, 36.3% share of total production between 2002 and 2014), polypropylene (PP, 21.0%), polystyrene (PS, 7.6%) and polyvinylchloride (PVC, 11.8%)^2^.

A strong body of evidence has shown that even the most recalcitrant plastics can be partly metabolized by the presence and activity of specific microorganisms, with several strains of degrading bacteria and fungi identified and described^3,4^. An important breakthrough was recently made in the case of polyethylene terephthalate (PET), with an efficient degrading enzyme isolated and characterized from *Ideonella sakainensis*, a bacterium isolated from a dumping site^5^, while for the most abundant plastic, PE, evidence of degradation still very limited (<10% for high-density polyethylene, HDPE), and the complete degrading mechanisms are still being investigated^6^.

The investigation of the microbial ecology of plastic materials is a fundamental step not only to isolate and characterize strains with potential degrading abilities, but also to understand the environmental impact of plastic materials in the environment. Studies have been mostly conducted on plastic waste collected from aquatic environments, and highlighted that the biofilm thriving on the surface of PE, PS and PP harbor distinct microbial communities from the surrounding water, and that the plastic type has also an influence on the microbial composition^7–9^. On the other hand, only a few studies have been carried out on soil environments, albeit recent evidence indicate that plastics are abundant in soils. In particular, it was estimated that more than 300,000 t of plastics are added annually every year to agricultural fields in Europe and North America, while analytical measurements highlighted concentrations of microplastics up to 6.7% in soils close to an industrial area^10^. Microplastics can also be transported along the soil profile by earthworms^11^ but their impact on soil quality ecology is still largely unknown. Machado *et al*.^12^ studied the effects of PE on soil biophysical properties, and found that the plastic decreased bulk density, and increased microbial activity. Interestingly, it was also found that PE can affect the impact and mobility of organic pollutants: for hydrophobic compounds such as polycyclic aromatic (PAHs) bioavailability and toxicity on microorganisms was reduced, as well as their biodegradation^13^, while for more polar pesticides sorption was reduced, with an increased mobility as compared to soil particles^14^.

Another important aspect to be considered in plastics pollution is its possible role as carrier of pathogenic bacteria: this wasn’t addressed in soil environment but only in aquatic environments, namely the North and Baltic Sea, where it was found that PE plastics had a selective enrichment of *Vibrio* species, including the pathogenic *Vibrio parahaemolyticus*^15^. On the other hand, several studies conducted on landfills and other terrestrial sites focused on the isolation and characterization of PE-degrading strains^16–19^, but a comprehensive assessment of the bacterial communities thriving on plastic wastes is to our knowledge still missing. In order to fill this gap, we have sampled different plastic wastes from a former landfill that has been closed more than 35 years ago. Samples were structurally characterized by Infrared (IR) and Raman spectroscopies, Differential Scanning Calorimetric (DSC) analysis, and inspected by Scanning Electron Microscopy (SEM) and X-ray Fluorescence (XRF), while at microbiological level Illumina-based amplicon sequencing and plate culture isolations were carried out to assess the structure of bacterial communities living on the plastics. Molecular analyses of bacterial communities were also conducted for comparison on the landfill soil and on a neighboring uncontaminated soil. The main hypotheses being tested in the work were: (i) different types of plastics select for different bacterial communities; (ii) structure of bacterial communities can be correlated with physico-chemical properties of the plastics, including the degradation degree.

## Results

### Plastics sampling and characterization

Five different plastic bag wastes were retrieved in replicates from landfill site in Northern Italy that has been abandoned since 1982. The plastic samples were characterized by their color and labelled as black (B), white (W), red (R), green (G) and yellow (Y). Three distinct films were retrieved for each colored plastic, and all microbiological and material analyses were performed in triplicates on these replicates.

**Figure 1:**
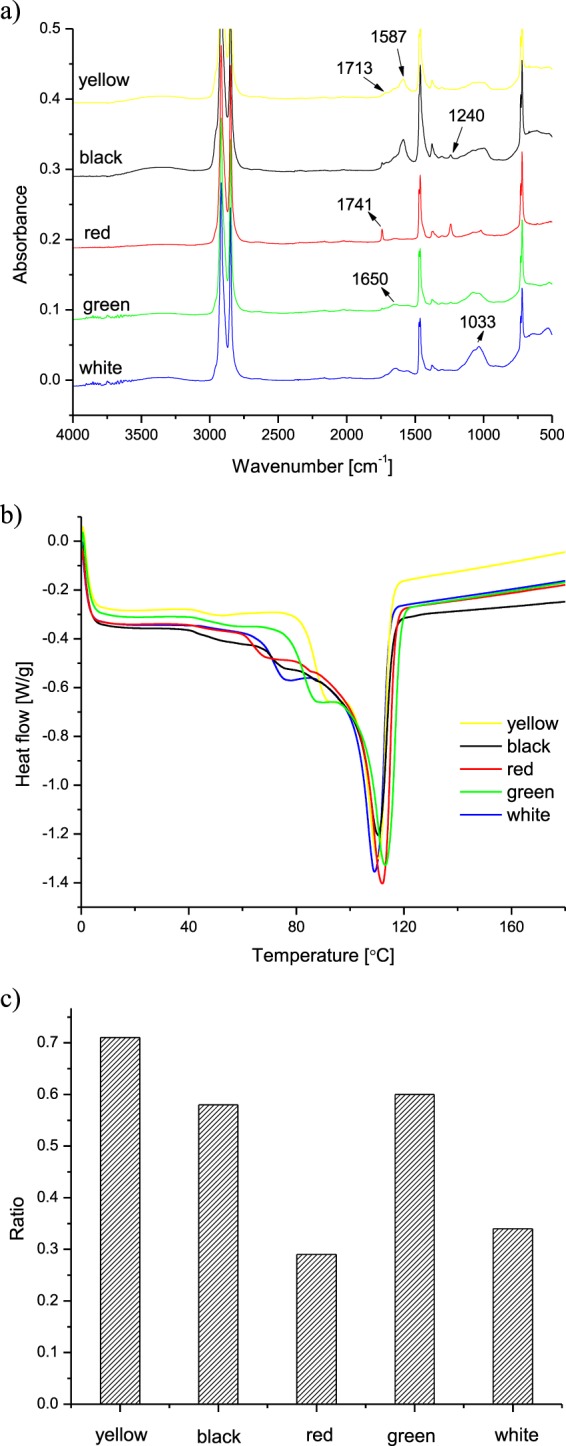
IR spectra (**a**) and DSC thermograms (**b**) of the analysed plastic samples.

Plastic samples were characterized by IR and DSC analyses. IR analyses (Fig. 1a) confirmed that all plastics analyzed were PE, since all samples showed typical IR absorbance bands of PE^20^: 2916 cm^−1^ (CH_2_ asymmetric stretching), 2848 cm^−1^ (CH_2_ symmetric stretching), 1473 and 1463 cm^−1^ (bending deformation), 1370 cm^−1^ (wagging deformation) and 730–720 cm^−1^ (rocking deformation). Other bands indicating degradation occurred: a broad band around 3300 cm^−1^ present in all the samples which can be assigned to –OH, and a band around 1030 cm^−1^ appears which can be assigned to C-O. Except the red PE sample, all the other samples showed a band at 1650 cm^−1^, which can be assigned to unsaturated C=C bonds. Furthermore, in yellow and black samples, carboxylic acids carbonyl absorbance band at 1714 cm^−1^ appeared; in red sample, esters carbonyl absorbance band at 1741 cm^−1^ occurred.

When analyzed by DSC (Fig. 1b), pure PE usually shows a single melting step around 110–120 °C. Besides this major melting step, all the samples showed a significant shoulder melting peak around 60–100 °C, due to the degradation. In order to quantify the degree of degradation of these samples, the enthalpies of melting in the DSC thermograms have been deconvoluted according to the lower should peak (60–100 °C) and the higher major peak (110–120 °C), and a ratio of enthalpies between the lower peak and the higher peak was obtained (Fig. 1c).

In order to identify the pigments inside the polymers, FT-Raman spectroscopy and X-ray fluorescence were applied. Raman spectra of the different films from shoppers are reported in supplementary Fig. S1, compared with a reference PE film without any pigment. In Table 1 the frequencies of the peaks that cannot be addressed to PE (by comparison with the reference spectrum) are reported for each colored plastic. According to frequencies and the actual color of the films, possible dye and pigments can be inferred; the spectral data from inorganic fillers in plastics were also analyzed to obtain some insights on the materials. As a first information, the peaks in the 100–700 cm^−1^ region of the Raman spectra suggest that, despite the different colors, at least white, yellow and red PE films contain TiO_2_ pigments, probably used as a matting additive for PE. TiO_2_ can be obtained in two main polymorphs in nature, that are rutile and anatase. These two are significantly different in the Raman features, and can be distinguished. Raman signals at 446 and 603 cm^−1^ are the typical fingerprint of rutile TiO_2_ form [http://rruff.info], whereas peaks at 143, 198, 396, 515 and 639 cm^−1^ are a clear fingerprint of anatase TiO_2_ phase (Table 1). According to this, the Raman patterns of the different plastics highlight the main presence of TiO_2_ in rutile form in white PE films and also a very relevant amount of it in the yellow PE film, whilst in red PE film the main peaks in the 100–700 cm^−1^ region are consistent with a very high amount of anatase TiO_2_ form. For yellow film, the appearance of a peak at 143 cm^−1^ (the most intense of the anatase Raman spectrum) suggests that a low amount of rutile form is also present.

**Table 1:** Main Raman bands identified for the colored plastics analysed. The possible compounds present are indicated according to the cited references.

Colour	Raman bands (cm^−1^)	Possible compound	Reference
White	446, 603	TiO2 rutile	www.RRUFF.info
Red	143, 198, 396, 515, 639, 721	TiO2 anatase	IRUG Spectral Database www.irug.org (Price & Pretzel, 2000)
Yellow	143, 446, 603, 724, 1003, 1251, 1400, 1528, 1595	Diarylide Yellow, Azo Dye + TiO2 + Titanox	IRUG Spectral Database www.irug.org (Price & Pretzel, 2000)
Black	1303, 1598	Carbon black	(Boccaleri *et al*., 2006)
Green	104, 202, 293, 515, 548, 646, 697, 709, 743, 779, 820, 959, 982, 1085, 1143, 1215, 1284, 1340, 1392, 1509, 1540	PG - Green Pigment Cu-Phthalocyanine	IRUG Spectral Database www.irug.org (Price & Pretzel, 2000)

For the black PE film the Raman spectrum is dominated by the broad thermal/fluorescent emission that often occurs when carbon black phases are present, coupled with two broad peaks at 1598 and 1301 cm^−1^ that identify this kind of black pigment^21^. Regarding green plastics, a very effective excitation was achieved using a red 633 nm He:Ne Laser, that evidently excites predominantly the Raman activity of the dye. On the basis of a search/compare procedure on web-based Raman spectral database IRUG^22^ the spectral profile was found to be consistent with an industrial green dye PG - Green Pigment Cu-Phthalocyanine (Table 1).

Once addressed the spectral features related to TiO_2_, in the spectrum of yellow PE film a series of peaks not related with PE modes can be found at 1003, 1251, 1400, 1528 and 1595 cm^−1^. Also in this case, these spectral features were compared with industrial yellow dyes in IRUG (Table 1), and a family of industrial additives, based on diarylide azo dyes was identified.

Though a very long acquisition (coadding 15000 spectral scans) was performed on the samples, the differences in signals for red plastic film did not allow the identification of spectral features helpful to identify the presence of a red dye or pigment. However, it is commonly reported that, while for yellow colors the use of organic compounds has several advantages especially in terms of thermal stability, for red coloring a very common way is the use of iron oxides, whose spectral features are very difficult to outline.

Further hints on the composition of PE fragments were obtained by elemental analysis using EDX (Supplementary Fig. S2). This technique was sided by a comprehensive overview on the samples at 500, 1000 and 2000x magnification ratios using backscattered primary electrons, that favored the comprehension of the topological distributions of chemically different species. EDX measurements carried on over a 1 mm^2^ area highlighted that, in addition to Raman information regarding the presence of TiO_2_ in white, red and yellow plastics, also in green and black samples this pigment is used, with the aim to mate the film appearance, with an evidently lower amount than in white film. With a further detail, the presence of traces of other metals was highlighted. In particular, as expected by the use of green phtalocyanine, a homogeneously distributed signal due to the presence of Cu was found in green plastic parts. For the red plastic pieces, it is evident, with respect to the other materials, the presence of iron, that is not systematically retrieved in the other films.

Going into detail of the images in backscattered electrons (Fig. S2), it appears evident on one side the presence of distributed particles of TiO_2_ in the matrices, but also the evidence that the different morphology of the holes due to the ageing is also related to different chemical distribution of elements. In white and red plastics, that appear heavily damaged by the bacteria, a relevant compositional contrast can be seen, due to the presence of heavier elements inside the holes/cavities left by the microorganism removal. However, the composition appears to be significantly different: in both cases the identification of elements like Na, Mg, P, Si, Ca suggest the presence of earth traces, but in the case of red plastics a significantly higher amount of iron is systematically found, and it is also retrieved in the particles near the edges of the cavities. This evidence seems to suggest that the presence of iron-based pigments in the red film could promote the interaction with earth bacteria. The elemental composition, however, focuses the difference in colour due to different organic dyes inside the polymeric matrix.

**Figure 2:**
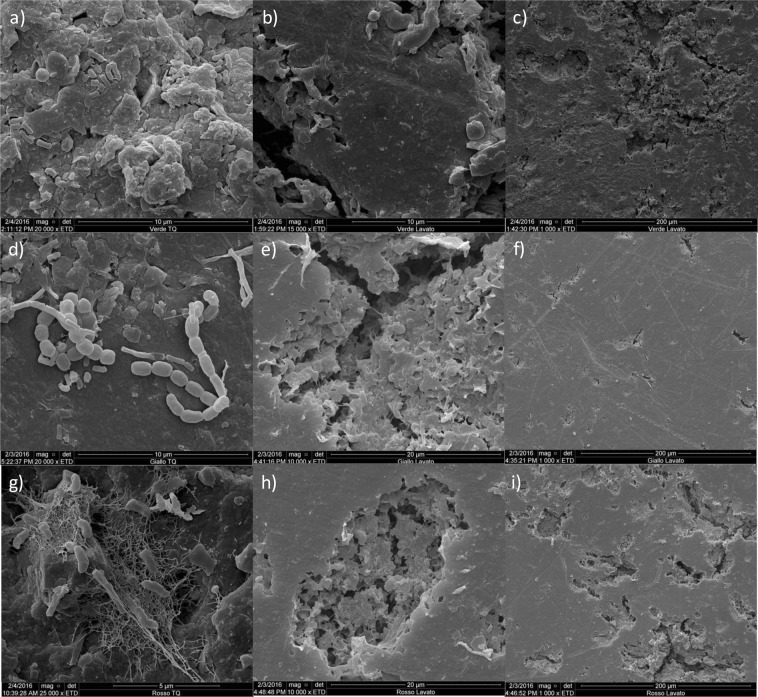
Scanning electron microscopy (SEM) images obtained with the gold coating technique; pictures show representative fields selected among all the observations performed.: (**a**) green plastic with biofilm; (**b**,**c**) green plastic after biofilm detachment; (**d**) yellow plastic with biofilm; (**e**,**f**) yellow plastic after biofilm detachment; (**g**) red plastic with biofilm; (**h**,**i**) red plastic after biofilm detachment.

Scanning electron microscopy was also applied to examine the plastics before and after the mechanical detachment of the biofilms: results are reported in Fig. 2 for the green (Fig. 2a–c), yellow (Fig. 2d–f) and red samples (Fig. 2g–i) as selected plastic. Per each, the first picture shows the plastic with the biofilm at 5 or 10 μm scale, the second the plastic without biofilm at 10 or 20 μm scale while the third column show the same plastic without biofilm at a lower resolution (200 μm). All plastics showed attachments of morphologically diverse rod-shaped prokaryotic cells, together with debris and filamentous structures that indicate a biofilm colonization (Fig. 2a,d,g). SEM pictures taken at similar scales after physical detachment indicated that the procedure was effective in removing the cells and biofilm from the surfaces (Fig. 2b,e,h). When the same samples were observed at a 200 μm scale a number of cavities were observed on the plastic surfaces, especially for the red (Fig. 2i) and the green (Fig. 2c) plastics, while the green plastic (Fig. 2f) and the white and black (pictures not shown) the surface was less characterized by cavities that can be related to degradation events.

### Molecular analyses of polyethylene-associated bacterial communities

Microbiological analyses were conducted both by culture-dependent and molecular methods, adopting a common approach for the retrieval of biofilms from the plastic samples.

For molecular analyses, DNA was extracted with FastDNA SPIN Kit for Soil and amplified in PCR with tagged primers targeting the V3-V4 regions of 16S rRNA. Illumina MiSeq sequencing of all amplicons resulted in a total of 454,043 high-quality filtered reads, which were downscaled to 235,212 after a rarefaction to a common number of 11,202 reads per sample. No samples were eliminated due to the downscaling. The average coverage was 90.1% ± 2.4, indicating that the sequencing effort was sufficient to describe the vast majority of bacterial communities in the samples. Of all analyzed sequences, 94% were correctly classified at the order level, 76.1% at the family, 60.5% at the genus and 30% at the species level. The total number of OTUs per sample varied between a minimum of 1,379 ± 22 for the white plastic to a maximum of 2,097 ± 7 for the red plastic; higher number of OTUs were detected in the soil samples, respectively 2,653 ± 115 for the dump soil where the plastics were retrieved and 2391 ± 54 in the neighboring uncontaminated soil.

**Figure 3:**
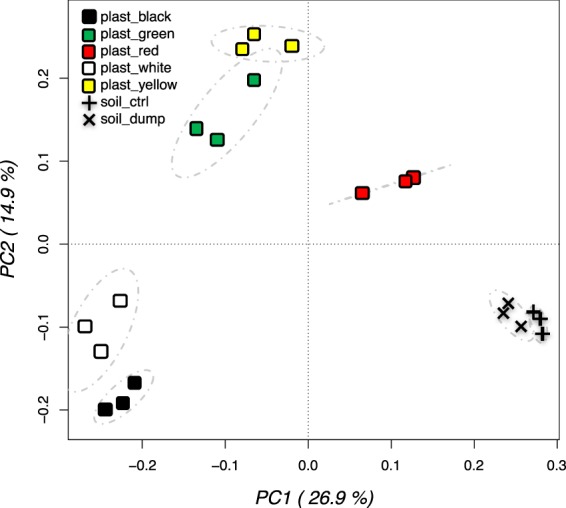
Principal component analysis performed on the total bacterial OTUs relative abundance data from plastic biofilms and soil. Replicates are labelled according to the samples.

A multivariate principal component analysis (PCA) performed on the relative abundances of all analyzed OTUs revealed a strong differentiation between samples (Fig. 3): each plastic hosted a different bacterial community, with the green and the yellow on one side, and the black and white on the other being closer one to the other. The red plastic bacterial community was found in-between the plastic and the soil samples replicates; finally, the latter were very close one to the other but still separated in two different groups (landfill and control soil).

**Figure 4:**
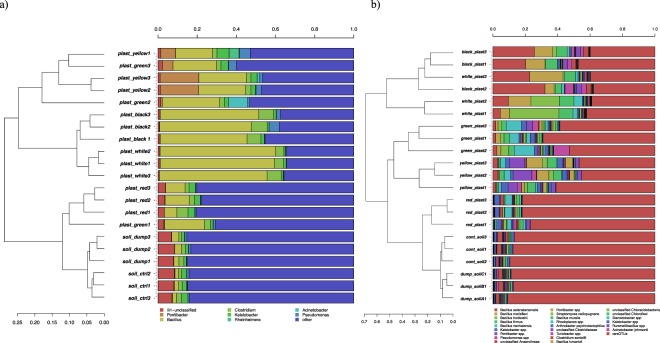
Hierarchical clustering of sequences classified at the genus level (**a**) or OTUs classified at species level. Bars of different colours indicate the different percentages of genera (**a**) or species (**b**) identified in each sample. Only taxa participating with >5% in at least one sample are presented for the genus level analyses (**a**), while for the OTU-level (**b**), a cut-off was applied to include OTUs covering the 95% of total diversity. Taxa with lower participation were added to the “other” groups.

This differentiation of bacterial communities was confirmed by hierarchical cluster analyses performed on sequences classified at the genus (Fig. 4a) and at the species level (Fig. 4b). In both cases the most abundant taxa, defined as those whose presence was >5% in at least one sample at least, are represented. In agreement with PCA results, the plastics communities clustered separately from the soil samples, and the 3 replicates from each sample (both plastic and soils) formed a separate cluster. A relevant exception was represented by the red plastic, that hosted a bacterial community that was closer to the soils than to the other plastics in terms of relative abundances of genera and species. Genus level analyses (Fig. 4a) revealed a significant enrichment of sequences classified as *Bacillus* on the plastic samples as compared to soil. In the control soil, an average of 1.9% of *Bacillus* sequences was detected: the relative presence of this genus increased to 3.2% in the dump soil and to 9.6% in the red plastic. Levels reached 22.4% in the yellow plastic, 24% in the green and then up to 47.2% and 57.5% in the black and white plastics respectively. Other genera significantly higher on plastic samples as compared to the soil were *Pontibacter* on the yellow and green plastics (15.6% and 2.2% respectively as compared to an average of 0.2% in the soils) and *Clostridium* in the black, white and red plastics (7.6%, 5.4% and 3.5% as compared to an average of 2.0% in the soils).

**Figure 5:**
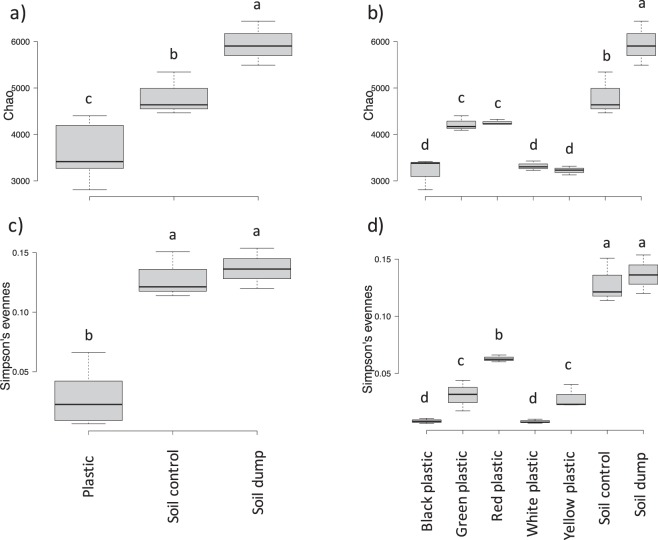
Chao diversity (**a**,**b**) and Simpson eveness indexes (**c**,**d**) of the bacterial communities of plastic and soil samples. Bar with the same letters are not statistically different according to Waller-Duncan test for comparison of means.

The clustering of the 24 most abundant OTUs is reported in Fig. 4b: 11 were classified at the species level, the rest were generally classified at the genus level. For the black and white plastics, these 24 OTUs represented up to the 60% of the total observed diversity; this was decreased to 50% in the yellow and 40% in the green plastics. On the contrary, these 24 OTUs covered the 20% only of the total bacterial diversity for the red plastics, which is a value quite close to the 18% found in the soil samples. This outcome clearly indicated that the plastic in soils were colonized by a few species becoming dominant, and it was confirmed by α-diversity indexes analyses (Fig. 5). When the comparisons were carried out between soil samples and plastics altogether, both Chao diversity (Fig. 5a) and Simpson evenness (Fig. 5c) were higher in the soil samples as compared to the plastics. Remarkably, the bacterial diversity in the dumpsite contaminated soil was significantly higher than in the neighboring uncontaminated soil (Fig. 5a). When plastic samples were differentiated according to the color, further significant differences in bacterial indexes were detected. The Chao diversity index (Fig. 5b) was higher in the green and red plastics, while the lowest index values were found for the black, the white and the yellow plastics. The situation was slightly different for the evenness as calculated with the Simpson index: the red plastic still had higher values than the other four, followed by the yellow and the green and afterwards by the black and white plastics. This result is in accordance with the PCA and clustering results presented above (Figs 3 and 4).

### A focus on bacilli as main colonizers of plastic wastes

**Figure 6:**
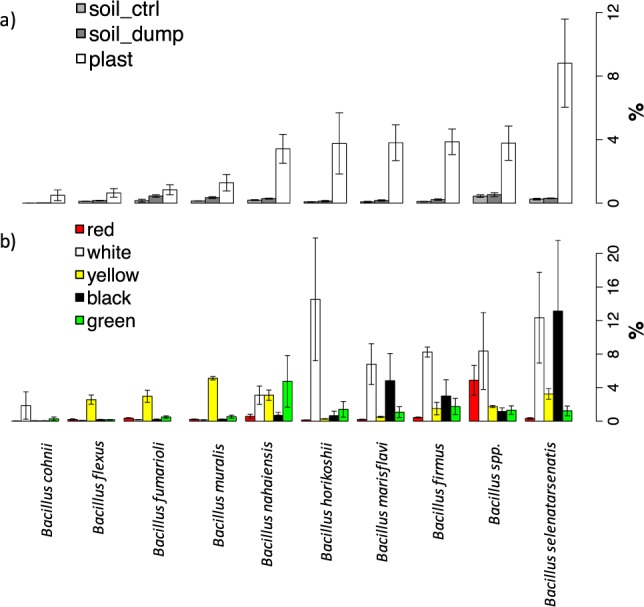
Relative percentages of the mostly abundant *Bacillus* species on the total bacterial community diversity. Data are presented by joining (**a**) or keeping separated (**b**) data from the 5 different plastic types.

Analyses of 16S PCR amplicons at the genus level (Fig. 4b) revealed that all plastic samples analyzed had high relative abundances of sequences classified as *Bacillus*, reaching a maximum of 57.5% of the total genera for the white plastics. For this reason, analyses were focused on the relative abundances of bacilli that could be identified and classified at species level. Results are presented in Fig. 6 for the 10 most abundant species detected: all except one (the second mostly abundant) were correctly classified at the species level. The results presented in the upper graph (Fig. 6a) confirmed that all species of *Bacillus* were significantly higher on the plastics as compared to the control and dump soil. The most abundant was *Bacillus selenatarsenatis*, followed by *Bacillus firmus*, *Bacillus marisflavi*, *Bacillus horikoshii*, *Bacillus muralis*, *Bacillus fumarioli*, *Bacillus flexus* and *Bacillus cohnii*. When the data were disentangled by plastic type, it was revealed that even the relative distribution of these very abundant *Bacillus* species was controlled by the color of the PE bags wastes (Fig. 6b). In particular, *B. selenatarsenatis* and B. *marisflavi* were more abundant on the white and black plastics, while *B. firmus*, B. *horikoshii* and *B. cohnii* dominated on the white plastics only. The yellow plastics had quite a different structure of *Bacillus* communities, with the dominant species being *B. muralis*, *B. fumarioli* and *B. flexus*. It is worth noting that the red plastics, having higher levels of degradation (Figs 1 and 2) and a bacterial community much closer to ones from the soils (Figs 3, 4 and 5) had also a much lower relative abundance of these *Bacillus* species.

### Inferences on functional genes abundances in the bacterial communities living on the plastic surfaces

A Picrust analysis on OTUs relative abundances was applied in order to infer the relative abundances of functional genes of interest in the bacterial communities thriving on the plastic surfaces as compared to the ones from soil samples. NSTI (Nearest Sequenced Taxon Index) values were 0.2466 ± 0.0003%, thus showing a very limited variability among replicates. Analyses were conducted at different KEGG reference hierarchy levels: results are reported in Table S1 for level 2, in Table 2 for level 3 and in Table S3 for single KEGG orthologs. Each table reports per each variable the p-values for two-sample comparisons between plastics and soil (only variables with p < 0.05 are reported), the average and standard deviation of relative % abundance. Variables with higher significant values in soils are highlighted in green, variables with higher significant values in plastics are highlighted in yellow. Concerning the major functional genes classes, plastics bacterial community had significantly higher levels of genes involved in cell motility, cellular processes and signaling, environmental adaptation, folding sorting and degradation, transcription and translation; on the contrary, soil communities were higher in amino acid, carbohydrate, terpenoids and polyketides metabolisms, cell communication, transport and catabolism, xenobiotic biodegradation and metabolism (Table S1). A deeper analysis at level 3 KEGG pathways (Table S2) highlighted indeed that soil communities had significantly higher levels of genes involved in the pathways for the degradation of atrazine, benzoate, bisphenol, dioxin, ethylbenzene, polycyclic aromatic hydrocarbons, styrene and xylene degradation. On the contrary, nitrotoulene degradation was the only xenobiotics degradation pathway enriched in plastic communities, together with other pathways of interest such as bacterial chemotaxis, bacterial motility, carbon fixation, electron transfer, germination, lipid metabolism, methane metabolism, plant-pathogen interactions, protein exports and kinases, secretion systems, signal transduction, sporulation and transcription machinery (Table S2).

**Table 2:** Identification of strains isolated from plastic samples and able to grow on paraffin as sole carbon source.

Strain	plastic origin	closest hit	S_ab score
UC7466	Yellow	*Bacillus pumilus*	0.992
UC7530	Yellow	*Arthrobacter sp*.	0.969
UC7508	Yellow	*Bacillus simplex*	0.984
UC7517	Yellow	*Bacillus muralis*	0.988
UC7534	Yellow	*Bacillus simplex*	1.000
UC7467	Yellow	*Pseudomonas alcaligenes*	1.000
UC7487	Black	*Bacillus aquimaris*	0.966
UC7476	Black	*Bacillus mycoides*	1.000
UC7520	Black	*Bacillus firmus*	1.000
UC7472	Red	*Pseudomonas plecoglossicida*	0.993
UC7473	Red	*Acinetobacter johnsonii*	0.998
UC7498	Red	*Comamonas testosteroni*	0.989
UC7450	Red	*Arthrobacter sp*.	1.000
UC7489	Green	*Pseudomonas thivervalensis*	1.000
UC7493	Green	*Bacillus boroniphilus*	0.971
UC7506	Green	*Bacillus megaterium*	0.997
UC7478	Green	*Paenibacillus woosongensis*	0.888
UC7503	Green	*Bacillus idriensis*	1.000
UC7509	White	*Bacillus firmus*	0.969
UC7533	White	*Bacillus firmus*	0.997
UC7494	White	*Bacillus luciferensis*	0.982
UC7531	White	*Lysinibacillus fusiformis*	0.751
UC7528	White	*Stenotrophomonas maltophilia*	0.997
UC7529	White	*Bacillus firmus*	1.000
UC7512	White	*Bacillus sp*.	0.949
UC7511	White	*Bacillus subtilis*	0.760
UC7521	White	*Bacillus drentensis*	1.000

### Cultivable bacteria isolated from the plastic surfaces

The same biofilm samples retrieved from the plastics and subjected to Illumina analyses of 16S rRNA PCR amplicons were used for the isolation of cultivable bacterial species. In order to focus not on the total bacterial community but on the species with affinities towards plastics, a selective minimal medium with paraffin as sole carbon source was employed, with the latter being a model molecule used to select strains with potential PE-degrading abilities. Decimal dilutions were plated, and representative colonies were picked, purified by three consecutives subculturing, dereplicated by RAPD analyses and finally identified by Sanger sequencing of PCR amplicons. The results reported in Table 2 show 27 different strains that were isolated from the 5 different plastic analyses: 6 from the yellow plastic, 3 from the black, 4 from the red, 5 from the green and 9 from the red. In agreement with the molecular analyses reported in Figs 4 and 6, *Bacillus* species dominated also the cultivable fraction, with 17 out of all 27 isolates belonging to this genus, with 12 different species identified (Table 2). The relative abundances of these species were also in agreement with the molecular data: *B. simplex* and *B. muralis* for the yellow plastics, *B. firmus* for the white and black. However, the most abundant *Bacillus* species according to the molecular data, *B. selenatarsenatis*, was not detected among the isolates. Other isolates of interest were *Pseudomonas alcaligenes*, *Pseudodomonas gelcossicida*, *Pseudomoans thivervalensis*, *Arthobacter* spp. and *Lysinibacillus fusiformis*.

## Discussion

Different types of plastics were sampled from a former landfill and were confirmed to be all PE by IR analyses. Since the landfill was abandoned more than 35 years ago, it is most probable that these samples have aged for a long time, but they were mostly visually intact, in accordance with previous reports on the recalcitrance of plastic materials in soil environments^23^. Most studies on the microbial colonization of plastic wastes were conducted on aquatic environments, and usually compared different types of plastics. In a landmark study, Zettler *et al*.^7^ compared PE and polypropylene wastes sampled in the North Atlantic Subtropical Gyre, and found that the bacterial communities thriving on them had a very different structure and lower diversity from the seawater, and that the two plastic types were very different one from the other in terms of bacterial species colonizing them. This outcome was further confirmed by studies performed on PE, PS and PET samples from the same Gyre^24^, and on PE and PS from the North and Baltic Sea^15^. As far as we know, no comparison of different types of PE was ever conducted, either in aquatic or terrestrial environments. Samples were characterized by different colors, hence containing different pigments, but also had different physicochemical properties that point to different degradation events, which could be of either abiotic or biotic origin.

All samples had indeed different IR and RAMAN spectra (Fig. 1a) and DSC and XRF results.

In particular it seems quite clear that the black and white samples have a similar thermal behavior with a family of chains that melt at a similar temperature (≈90 °C), while yellow and green samples have another first melting peak (≈80 °C). The red sample seems different to the other for many reasons: the thermal behavior (1° Tmelt ≈70 °C), the presence of a carbonyl group on the surface (IR spectra), the rutilo TiO2 form (Raman) and the presence of iron (SEM-EDS). All these analyses are in agreement with the OTUs PCA results.

SEM inspections were carried out before cells detachment (Fig. 2a,d,g) revealed a complex biofilm colonization of the different PE plastics, with cell morphologies, gel matrixes and hyphal-like structures, while after cells detachment (Fig. 2b,c,e,f,h,i) it was evident that the surfaces of the different polymers were partly degraded with cavities and holes. Biofilms on PE are in line with SEM analyses previously carried out on plastic waste from aquatic environments^8,25,26^, while the signs on the surfaces are similar to those identified on PE polymers after degradation experiments^27–29^. These results, together with the IR and DSC analyses indicate that after several years in the landfill soil, all plastic wastes here studied underwent a partial surface degradation.

Illumina molecular analyses clearly indicate that each plastic harbored a different bacterial community from the other, starting from the phylum level (data not shown) up to the genus (Fig. 4a) and the species level (Fig. 4b). As far as we know, this is the first report showing that different PE wastes from a soil environment harbor different bacterial communities. A common feature is the predominance of *Bacillus*, ranging from a minimum of 10% on the red plastic to more than 50% on the white. This outcome is in agreement with evidence from Nowak *et al*.^30^, who compared the colonization of microorganisms from different soils incubated for 225 days with films of pure LDPE, pure polyester and a PE-polyester mixture and found a predominance of species from this genus, in particular *B. cereus*, *B. amyloliquefaciens*, *B. pumilus* and *B. mycoides*. The latter two species were indeed also found in our work as isolates respectively on the black and yellow plastics (Table 2) while the Illumina based analyses (Fig. 6) revealed a higher percentage of other *Bacillus* species. The most abundant, reaching more than 10% of the total bacterial community on the white and black plastics was *B. selenatarsenatis*, a strain isolated from a glass manufacture plant, whose genome has recently been sequenced^31^. Notably, this species is known to reduce arsenic and selenium: we don’t have evidence of a higher concentration of these elements in the soil and plastics analyzed, but a hypothesis might be that the reduction of these or other elements can be coupled to the oxidation of PE. An investigation of the reference genome deposited in GenBank (accession number BASE01000001) reveals indeed that this species harbors several oxidase genes, including a multicopper oxidase that in the PE-degrading strain *Rhodococcus ruber* has been linked to the degradation of the polymer^32^. Among the other species, *B. firmus* was found to degrade several pollutants including Fipronil^33^ and dyes such as methylene blue^34^. Degradation of colorimetric dyes different from the ones identified here, namely Congo red and methylene blue, was also reported for *B. marisflavi*^35^, a species which in our samples was most abundant in the white, black plastics and to a lower extent in the green, yellow and red. These results point to a possible degradation activity towards not only the PE itself but also towards the dyes present in the different colored plastics studied here. However, several strains including *Bacillus* species were also isolated with cultivation techniques using paraffin as a sole carbon source, which is a model molecule for PE utilization, thus pointing to a dual utilization. Furthermore, it should also be taken into account that the bacterial species colonizing the PE wastes can also retrieve nutrients from the soil particles.

Alfa-diversity analyses revealed that the soil samples had a higher diversity as compared to the plastic samples (Fig. 5): this result different from evidence from aquatic environments where the richness was higher on the plastics as compared to the surrounding seawater. Here we also found higher evenness on plastics as compared to soils, while in sea the contrary was found^7^. A study on plastics contamination in an urban river found higher bacterial diversity and richness on organic matter as compared to the plastics surface^36^. Interestingly, we also found that the dumpsite soil had a higher Chao richness as compared to the control neighboring soil: this may be explained by the intermediate disturbance hypothesis of Connell^37^, which states that perturbation events that are not deleterious for the majority of the populations can reduce dominance and promote diversity. Originally proposed for plant ecology studies, it was afterwards validated also in microbial ecology^38,39^.

An inference on the relative abundances of functional genes between the bacteria thriving on the plastic surfaces and the ones in the soil was explored by Picruts analysis. As far as we know, the only similar analysis was conducted by Debroas and colleagues on bacterial communities from PE, PET and PS plastic wastes of the North Atlantic subtropical gyre^24^. Our results are quite in contrast, since we had in common only an increase in cellular processes, signaling and transport genes, while several major classes such as xenobiotics biodegradation, transport and catabolism, lipid metabolisms were in our case lower in the plastic communities, while higher in the above cited study. A possible explanation is due to the fact that aquatic environments host a much lower diversity than soils, hence an enrichment of several functional genes can be expected on the plastics from the ocean. The downregulation of several genes related to xenobiotics degradation (Tables S1 and S2) can also be explained by the higher microbial abundances in the soil environment, but also by the fact that genes involved in PE degradation are still mostly unknown, and hence not categorized in the KEGG database used by PICRUST analysis.

The evidence here presented indicate that terrestrial PE plastic wastes harbor very different bacterial communities from the surrounding soils, and that factors such as the presence of dyes and the degradation levels also influence the type of bacterial assemblage present. As already shown for aquatic environments^24^, PE has the ability to select for specific bacterial taxa also in soils, including xenobiotic degraders and genera comprising several pathogens such as *Bacillus*. Further research should be conducted in order to better understand the impact of terrestrial plastic wastes also from a microbiological and ecotoxicological point view.

## Materials and Methods

### Site description and samples collection

Sampling was carried out in June 2015 in an abandoned dumpsite in Northern Italy (Località Tavernelle, Fiorenzuola d’Arda, Piacenza Province). The dumpiste had an area of 10,600 m^2^ and received an average of 12,000 m^2^ each year of municipal solid wastes between 1972 and 1982. In total, approximately 42,000 tons of wastes were grounded in 10 years up to a depth up to 4 m. While most organic materials and pollutants were either degraded or leached over years, several persistent plastic materials were found at the surface of the site during the sampling.

Sampling was carried out with aseptic techniques, retrieving from different part of the field 5 plastic films characterized by different colors: black (B), white (W), red (R), green (G) and yellow (Y). Three replicates per each plastic type were sampled, for a total of 15 replicates. Furthermore, soil samples from the dumpsite and from a neighboring uncontaminated site (cultivated with *Medicago sativa* L.) were also collected in triplicates. All samples were immediately brought to the lab and analyzed.

### Recovery of biofilms from plastic samples

In order to retrieve the biofilms growing on the surface of the plastic samples, the films were firstly aseptically cleaned from soil debris, cut in squares of 10*10 cm, and homogenized with 90 mL of sterile physiological solution using a Stomacher machine (400 Circulator; International PBI, Milan, Italy) for 1 minute at maximum speed for 5 minutes. The procedure was repeated three times. After extraction, the water phase containing the biofilms was centrifuged at 4000 rpm for 5 minutes at 4 °C, and resuspended in 5 mL of physiological solution. An aliquot of the resuspension was then used for DNA extraction and concomitant molecular analyses, and another one for culture-based analyses as explained below.

### Plastic samples characterizations

All analyses on plastic samples were carried out after the biofilm retrieval process described above.

Attenuated Total Reflectance (ATR) spectra were obtained using a diamond crystal on a Perkin Elmer Frontier instrument, with 32 scans per replicate in a scanning range of 4000–500 cm^−1^ and scan rate of 4 cm^−1^. Differential Scanning Calorimetric (DSC) analyses were performed on 5–6 mg samples using a TA Q20 instrument in hermetic aluminium pans, under nitrogen flow (50 mL/min) at 10 °C/min heating rate from 0 to 200 °C.

Scanning Electronic Microscopy (SEM) analyses were performed on 0.5 cm^2^ plastic samples stepwise dehydrated in ethanol 75%, 85%, 95% and 100% for 1 h at room temperature. Critical point drying was performed in a BalTEC CPD030 critical point dryer. Samples were then gold-coated^40^ and observed with a Philips XL30 ESEM (Environmental Scanning Electron Microscope).

Raman spectra were collected on a Bruker RFS100 FT-Raman spectrophotometer, equipped with a Nd^3+^:YAG laser (1064 nm) and a N_2_ cooled Ge detector. Spectra were collected for 15000 scans at 4 cm^−1^ with a laser power between 70 and 100 mW depending on the sample.

For the green sample, spectra were collected on a Jobin Yvon Horiba Labram HR micro Raman spectrophotometer using a He:Ne laser (633 nm) and a Peltier cooled matrix detector. Spectra were collected using a 1800 lines/mm 50x objective and acquiring the spectra for 30 seconds with a 10 repetition per spectral region.

Backscattered electron images and EDX analyses were collected on a Quanta 200 FEI Scanning Electron Microscope.

### Isolation and characterization of cultivable bacteria

The biofilm retrieved from the plastic surfaces were plated by serial dilution on minimal salt medium (SM) containing (per L of distilled water): 1.0 g NH_4_NO_3_, 0.2 g MgSO_4_·7H_2_O, 1.0 g K_2_HPO_4_, 0.1 g CaCl_2_·2H_2_O, 0.15 g KCl, 0.1 g yeast extract (Difco) and 1.0 mg of each of the following microelements: FeSO_4_·6H_2_O, ZnSO_4_·7H_2_O and MnSO_4_^40^. Paraffin oil (2%) was added to the plates as sole carbon source in order to isolate strains with potential degradation abilities. Plates were incubated at 30 °C in aerobic conditions, and representative colonies from the different plates were chosen on the basis of morphological differences.

DNA from isolates purified colonies was extracted with Microlysis kit (Microzone, Haywards Heath, UK) according to the manufacturer’s protocol. Randomly Amplified Polimorphic DNA-Polymerase Chain Reaction (RAPD-PCR) was performed on each isolate using single stranded oligonucleotide primers RAPD2 (5′-AGC AGG GTC G-3′)^41^ and M13 (5′-GAG GGT GGC GGT TCT-3′)^42^. Amplification reactions with both primers were conducted in a programmable T100 thermocycler (BioRad) in volumes of 25 μL containing 1 μM of primer, 3 mM of MgCl_2_, 0.2 mM of DNTPs, 2.5 U of Taq DNA polymerase (Fermentas, Selangor, Malaysia). The PCR fragments electrophoretic profiles were captured and patter analysis was performed with the Fingerprinting II software (BioRad) in order to obtain similarity profiles of bands based on Unweighted Pair Group Method with Arithmetic Mean (UPGMA) and select representative unique isolates. The latter were identified by PCR amplification using the primers P0 5′-GAG AGT TTG ATC CTG GCT-3′) and P6 (5′-CTA CGG CTA CCT TGT TAC-3′) as previously described^43^. The PCR products were purified using the Wizard SV Gel and PCR Clean-Up system, according to the package insert (Promega Corpo- ration, Madison, WI) and sequenced at the BMR Genomics of Padova, Italy. The identification of sequences was performed by alignment against the Ribosomal Database Project (RDP) database using the Naïve Bayesian Classifier^44^.

### Bacterial communities’ molecular analyses

Microbial DNA extraction was carried out from 0.5 mL of biofilm suspensions using the FastDNA SPIN Kit for Soil (MP Biomedicals, Solon, OH, USA) according to the manufacturer’s protocol, and the concentrations of double-stranded DNA in the extracts was determined using the Quant-iT dsDNA HS assay kit and the Qubit fluorometer (Invitrogen, Carlsbad, CA, USA).

PCR amplification of the bacterial V3-V4 16S rRNA hypervariable regions was carried out with the Phusion Flash High-Fidelity Master Mix (Thermo Fisher Scientific, Inc., Waltham, MA, USA) and the primer pairs 343F (5′-TACGGRAGGCAGCAG-3′) and 802R (5′-TACNVGGGTWTCTAATCC-3′), as previously detailed^45^. Amplifications were carried out in 25 μl of volume reactions, containing 1 ng of DNA and 0.25 µM of each primer, using a two-step PCR approach^46^. First step consisted in a 5 min step at 94 °C of initial template denaturation and polymerase activation, 25 cycles parted by 30 sec of denaturation (94 °C), 30 sec of primers annealing (50 °C) and 30 sec of primers elongation (72 °C), followed by a final elongation step (72 °C) of 10 min. The same conditions were applied in the second step PCR, where 1 µL of first step products served as a template was used and the same PCR conditions described were applied, using 8 cycles instead of 25. In order to allow simultaneous analyses of several samples in a single sequencing run, the second PCR step was performed using indexed forward primers throughout a 9 nucleic acids base extension at their 5′ end, with the first seven bases served as a sample index sequence for multiplexing, while the two bases next to the original primer served as linker bases, not matching any bacterial sequence at these position according to Ribosomal Database Project (RDP) entries.

High-throughput sequencing data filtering, multiplexing and preparation for concomitant statistical analyses were carried out as previously detailed^47,48^. In summary, paired-reads were assembled to reconstruct the full V3–V4 amplicons with the “pandaseq” script^49^ allowing a maximum of 2 mismatches and at least 30 bp of overlap between the read pairs. Samples demultiplexing was then carried out with the Fastx-toolkit (http://hannonlab.cshl.edu/fastx_toolkit/).

Mothur v.1.32.1^50^ was applied in order to remove sequences with large homopolymers (≥10), sequences that did not align within the targeted V3-V4 region, chimeric sequences^51^ and sequences that were not classified as bacterial after alignment against the Mothur version of the RDP training data set. The resulting high-quality sequences were analysed with Mothur and R^52^ following two main approaches: the operational taxonomic unit (OTU) and the taxonomy-based approach. For the OTU approach, sequences were first aligned against the SILVA reference aligned database for bacteria^53^ using the NAST algorithm and a kmer approach^54,55^, and then clustered at the 3% distance using the average linkage algorithm. OTUs having a sum of their abundances across all samples of than 0.1% of the total were grouped into a single “rare OTUs” group. For the taxonomy based analyses, sequences were classified into taxa using an amended version of the Greengenes database^56^.

Statistical analyses on OTU and taxonomy matrixes were performed in Mothur and R, and included hierarchical clustering with the average linkage algorithm at different taxonomic levels, Principal component analysis (PCA) to assess the unconstrained samples grouping, Canonical correspondence analyses (CCA) to assess the significance of different treatments on the analysed diversity. Metastats^57^ was applied to identify features that were significantly different between treatments. Functional genes were inferred from 16S rRNA data using PICRUSt^58^ and afterwards analyzed and visualized with STAMP^59^.

## Supplementary information

Supplementary figures S1 and S2

Table S1

Table S2

Table S3
