# Supplementary material for: Selective bacterial colonization processes on polyethylene waste samples in an abandoned landfill site
Source: Sci Rep. 2019 Oct 2;9:14138. doi: 10.1038/s41598-019-50740-w (PMC6775442; doi:10.1038/s41598-019-50740-w)
Supplement: Supplementary file 1 — Supplementary figures S1 and S2 [file 41598_2019_50740_MOESM1_ESM.pdf]

SUPPLEMENTARY INFORMATION

Title

Selective bacterial colonization processes on polyethylene waste samples in an abandoned landfill site

Authors

Edoardo Puglisi, Francesco Romaniello, Serena Galletti, Enrico Boccaleri, Alberto Frache, Pier Sandro Cocconcelli

Figure S1. Raman spectra of the different colored films as compared to a reference PE film (black spectral lines) without any pigment: a) yellow film vs reference PE; b) yellow film vs reference PE; c) black film vs reference PE; d) red film vs reference PE; e) green film vs reference PE.

a)

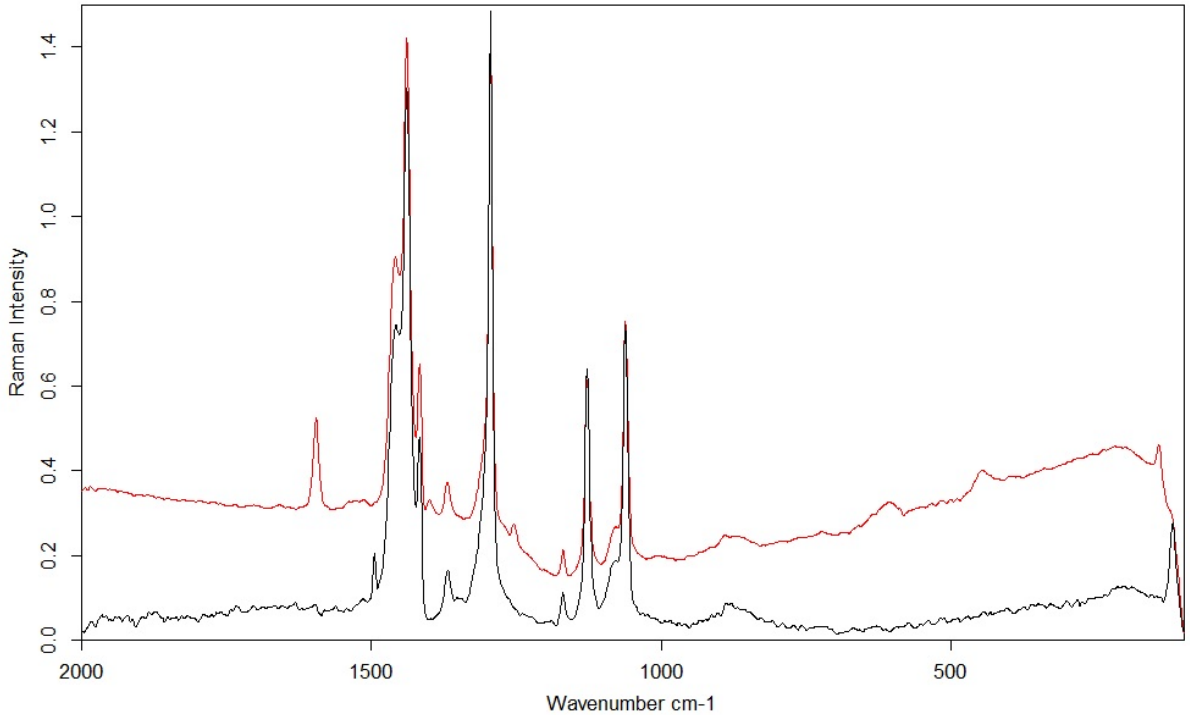

b)

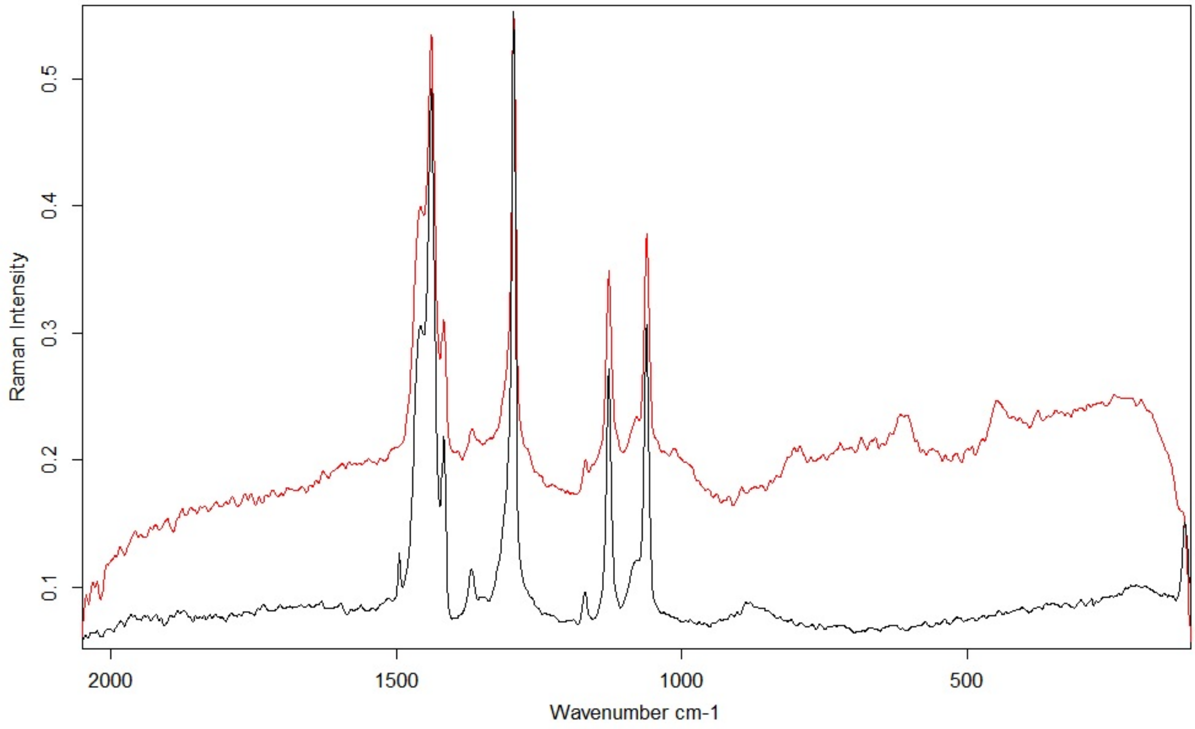

c)

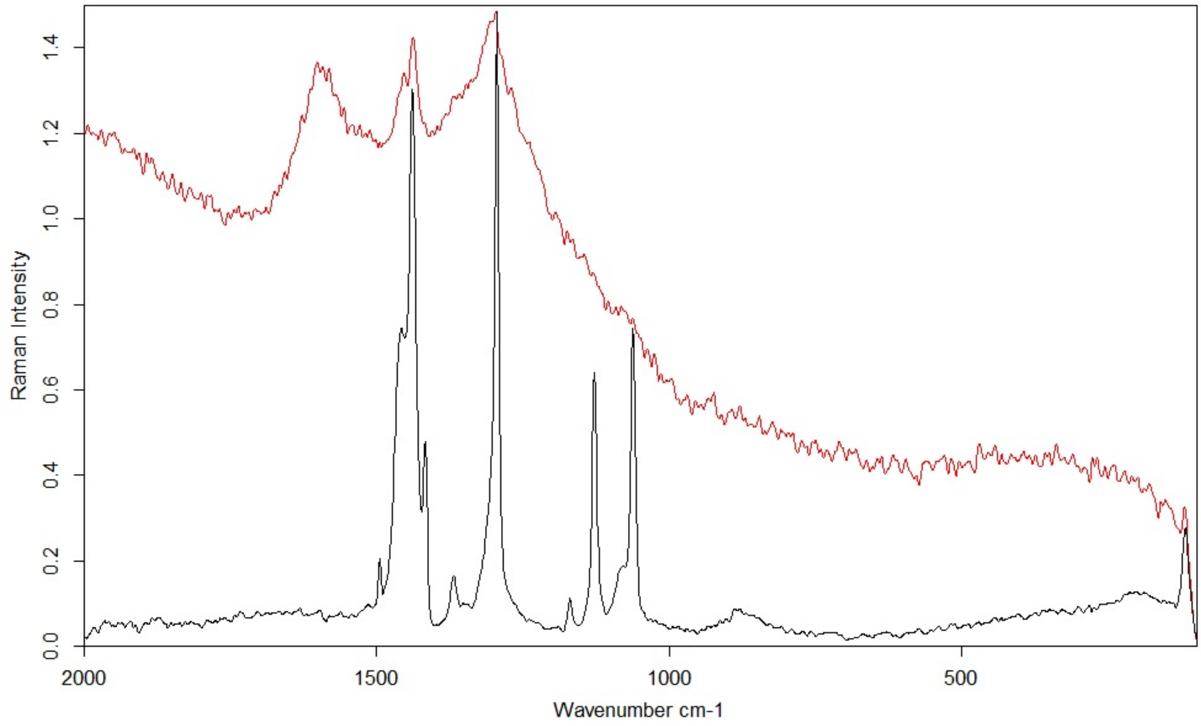
Raman Intensity
0.0
0.2
0.4
0.6
0.8
1.0
1.2
1.4
2000
1500
1000
500
Wavenumber cm-1

d)

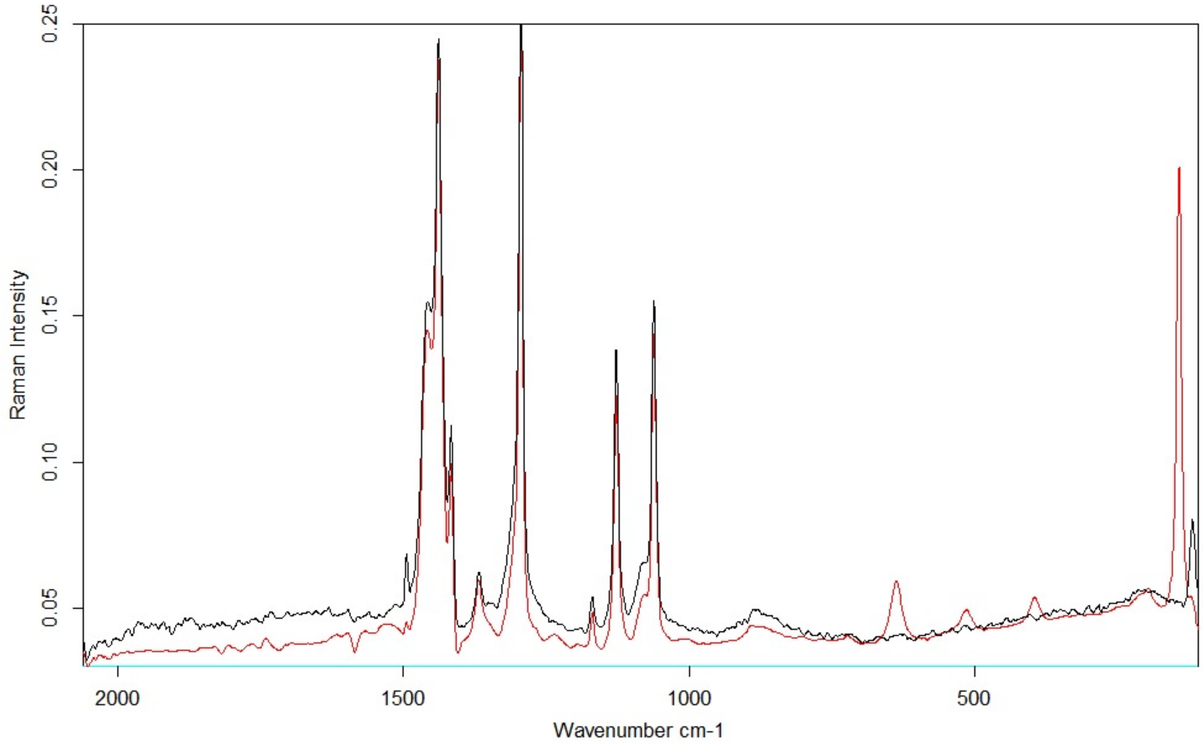
Raman Intensity
0.05
0.10
0.15
0.20
0.25
2000
1500
1000
500
Wavenumber cm-1

e)

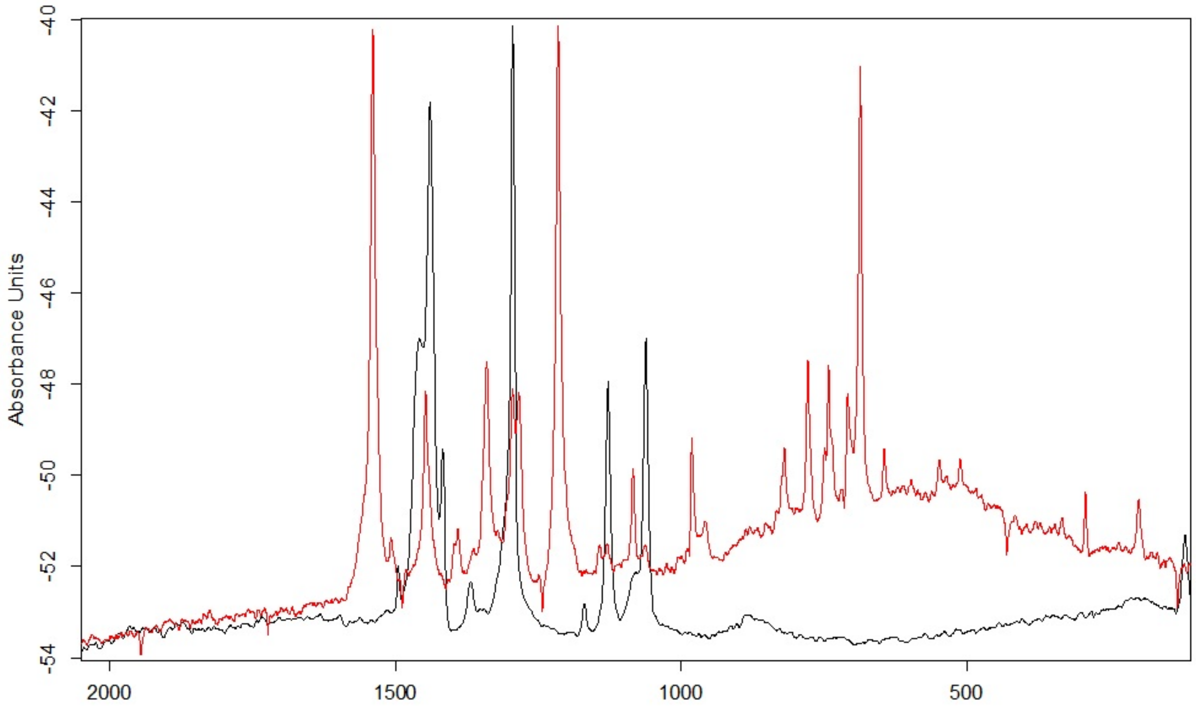

Figure S2. Scanning Electron Microscopy coupled to Energy-Dispersive X-Ray analysis (SEM-EDX) to assess the topological distribution of chemically different species on the plastic films without bacterial biofilm. Magnification ratios were applied at 500X (a), 1000X (b) and 2000X (c).

a)

White	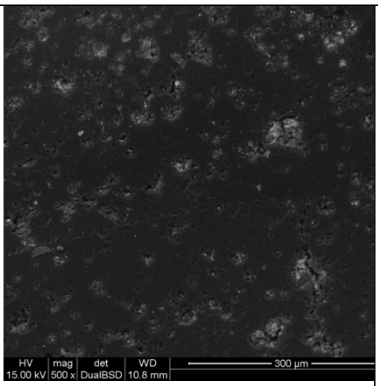	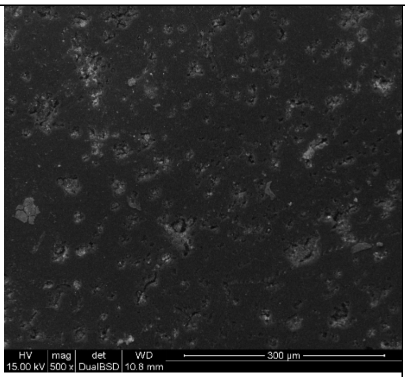	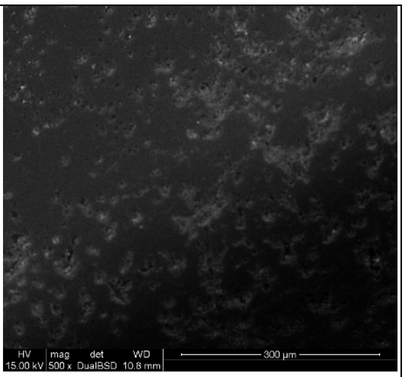
Yellow	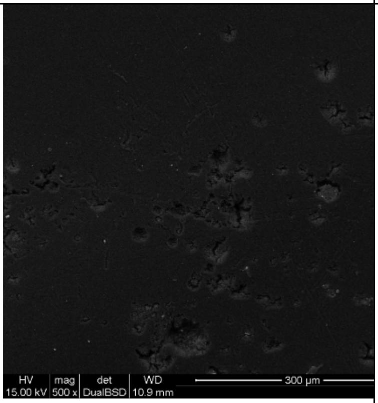	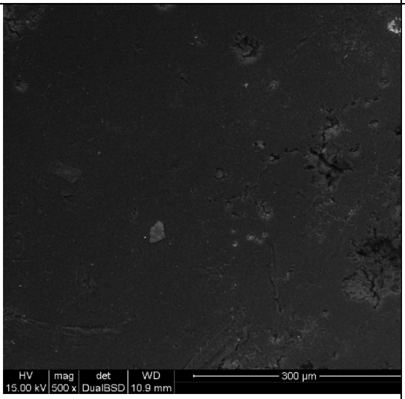	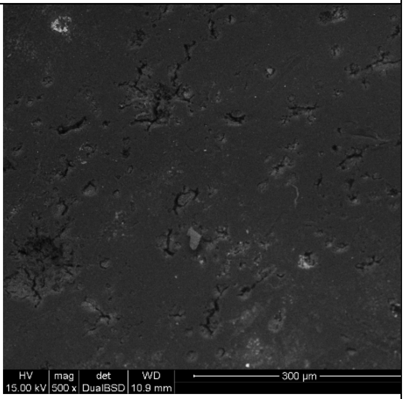
Red	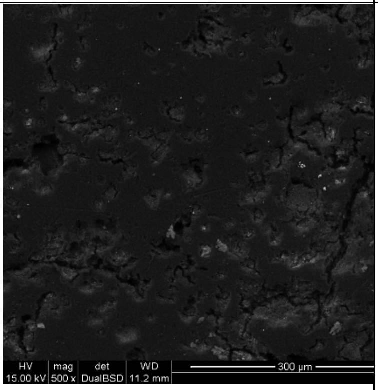	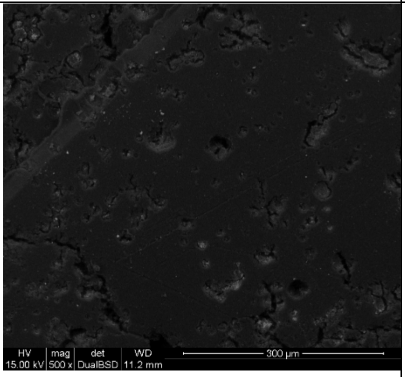	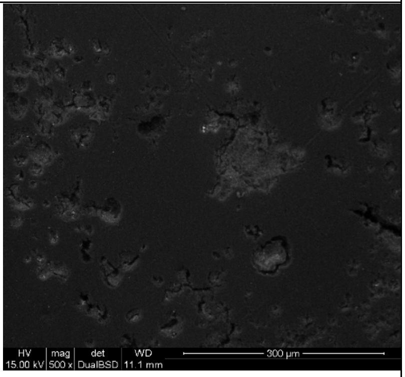

Green	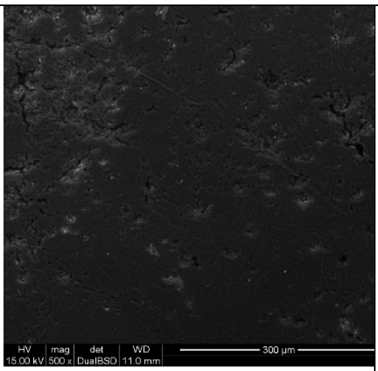	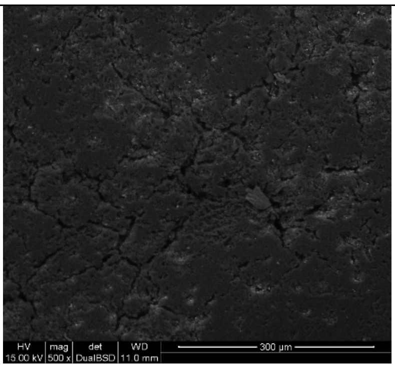	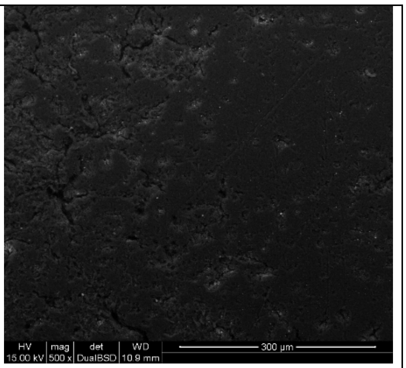

b)

White	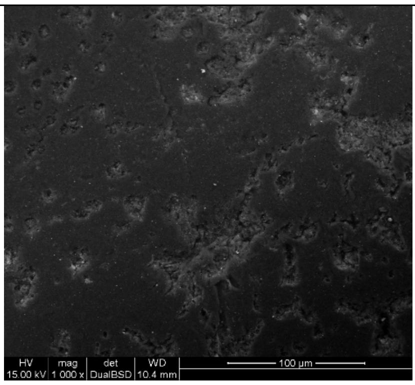	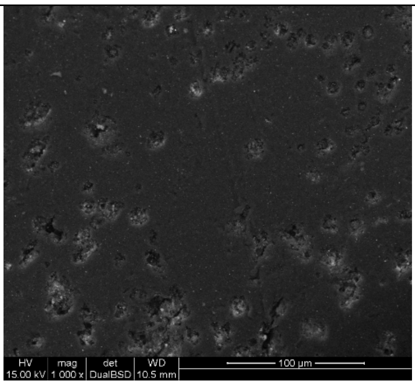	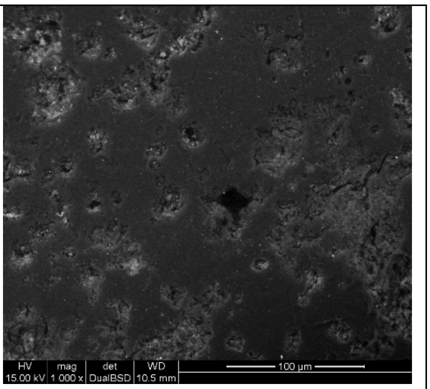
Yellow	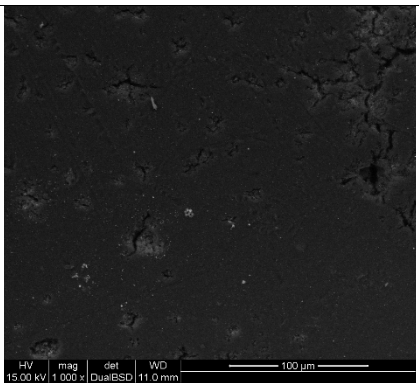	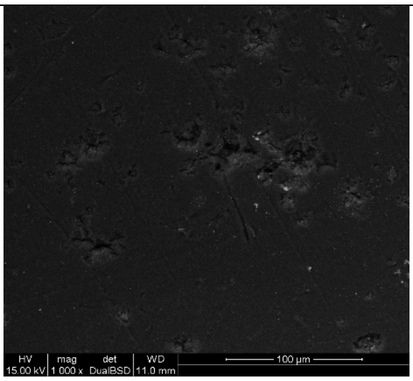	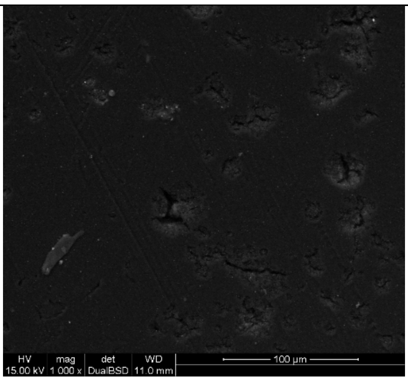
Red	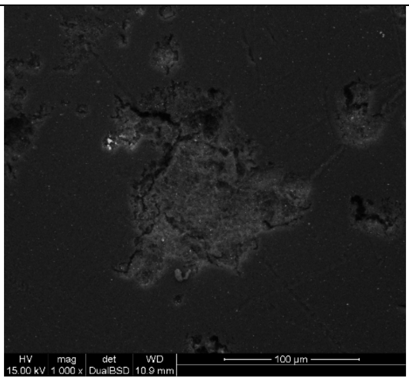	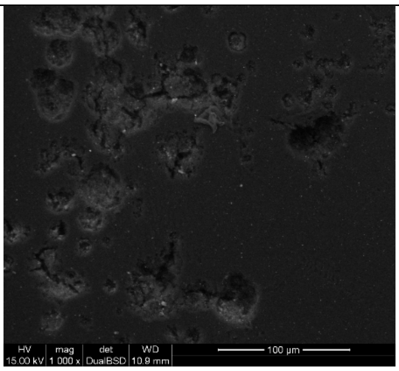	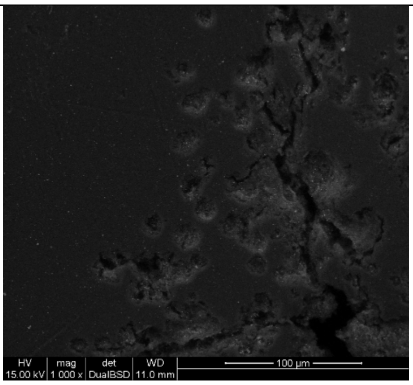

Green	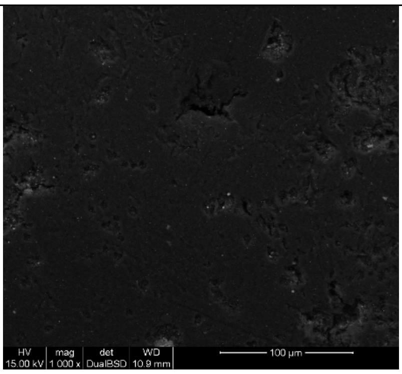	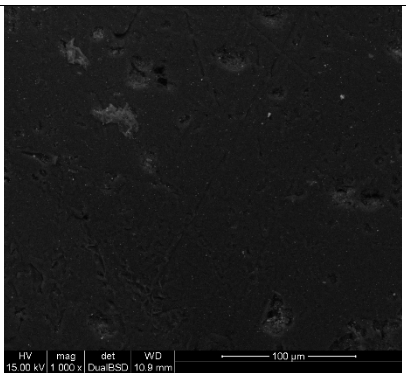	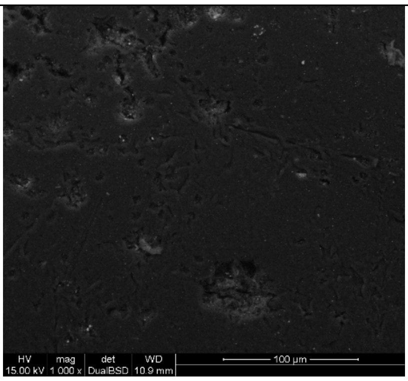

c)

White	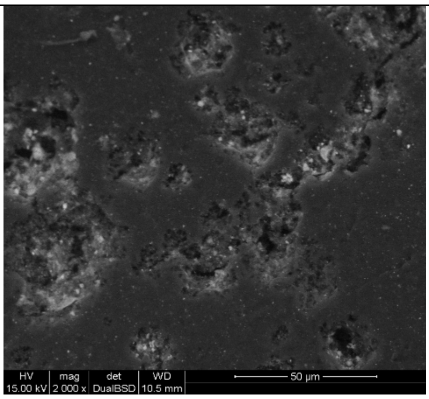	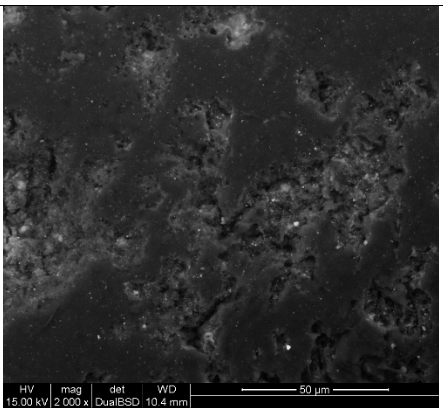	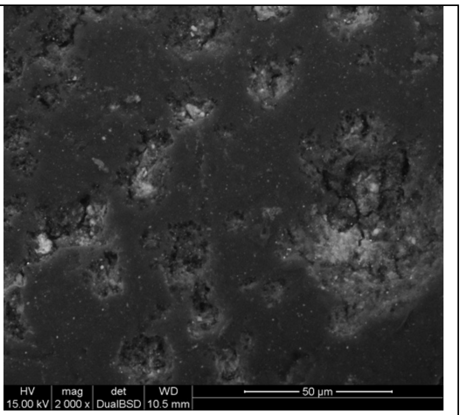
Yellow	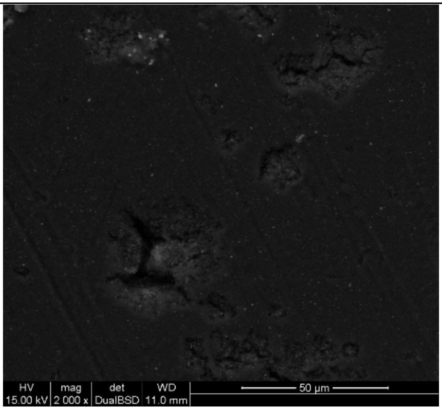	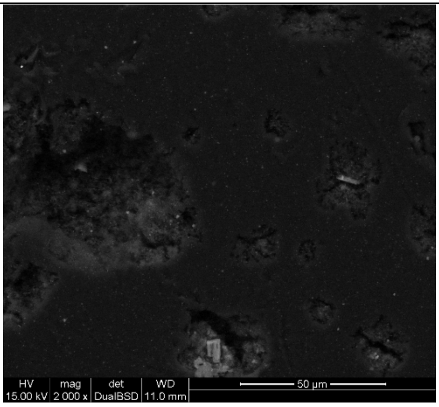	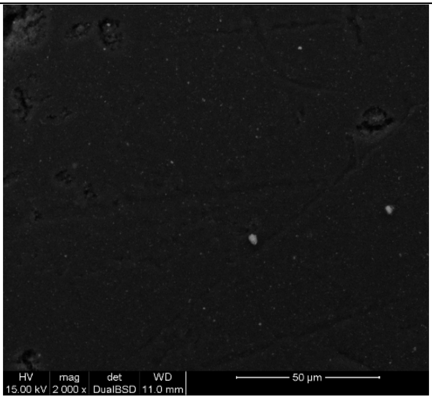

Red	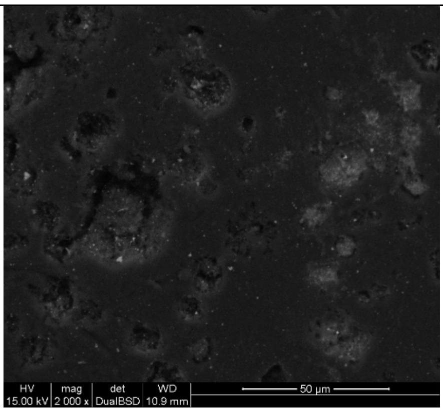 HV mag det WD 50 µm 15.00 kV 2 000 x DualBSD 10.9 mm	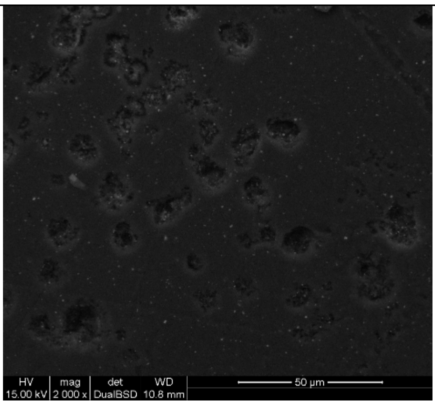 HV mag det WD 50 µm 15.00 kV 2 000 x DualBSD 10.8 mm	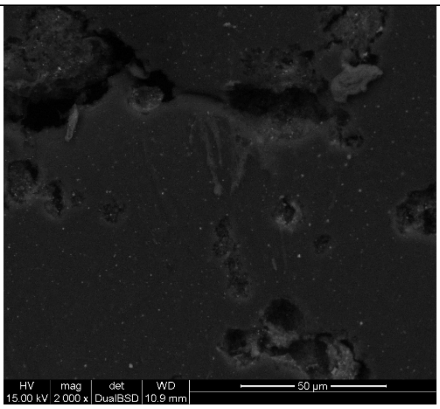 HV mag det WD 50 µm 15.00 kV 2 000 x DualBSD 10.9 mm
Green	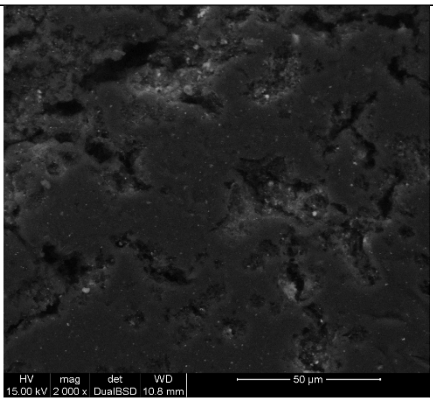 HV mag det WD 50 µm 15.00 kV 2 000 x DualBSD 10.8 mm	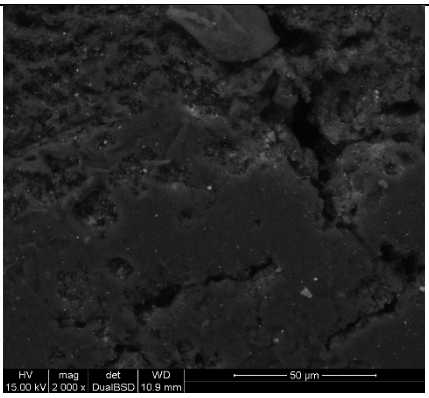 HV mag det WD 50 µm 15.00 kV 2 000 x DualBSD 10.9 mm	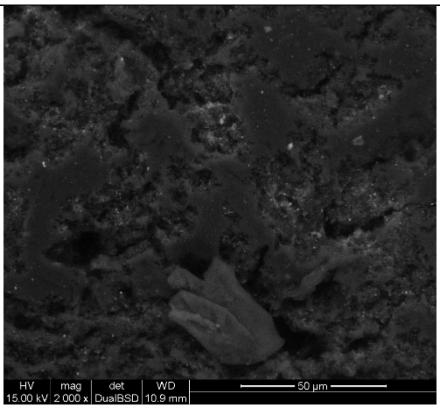 HV mag det WD 50 µm 15.00 kV 2 000 x DualBSD 10.9 mm
